# Ethical dilemmas posed by mobile health and machine learning in psychiatry research

**DOI:** 10.2471/BLT.19.237107

**Published:** 2020-02-25

**Authors:** Nicholas C Jacobson, Kate H Bentley, Ashley Walton, Shirley B Wang, Rebecca G Fortgang, Alexander J Millner, Garth Coombs, Alexandra M Rodman, Daniel D L Coppersmith

**Affiliations:** a Department of Biomedical Data Science and Psychiatry, Geisel School of Medicine, Dartmouth College, Suite 300, Office # 333S, 46 Centerra Parkway, Lebanon, NH 03766, United States of America (USA).; b Department of Psychiatry, Massachusetts General Hospital–Harvard Medical School, Cambridge, USA.; c Department of Statistics, Harvard University, Cambridge, USA.; d Department of Psychology, Harvard University, Cambridge, USA.

## Abstract

The application of digital technology to psychiatry research is rapidly leading to new discoveries and capabilities in the field of mobile health. However, the increase in opportunities to passively collect vast amounts of detailed information on study participants coupled with advances in statistical techniques that enable machine learning models to process such information has raised novel ethical dilemmas regarding researchers’ duties to: (i) monitor adverse events and intervene accordingly; (ii) obtain fully informed, voluntary consent; (iii) protect the privacy of participants; and (iv) increase the transparency of powerful, machine learning models to ensure they can be applied ethically and fairly in psychiatric care. This review highlights emerging ethical challenges and unresolved ethical questions in mobile health research and provides recommendations on how mobile health researchers can address these issues in practice. Ultimately, the hope is that this review will facilitate continued discussion on how to achieve best practice in mobile health research within psychiatry.

## Résumé

L'application des technologies numériques à la recherche psychiatrique entraîne rapidement de nouvelles découvertes et capacités en matière de santé mobile. Cependant, la multiplication des opportunités de recueillir passivement d'immenses quantités d'informations détaillées sur les participants aux études combinée aux progrès des techniques statistiques permettant aux modèles d'apprentissage automatique de traiter de telles informations a soulevé de nouveaux dilemmes éthiques concernant l'obligation des chercheurs: (i) de surveiller les effets indésirables et d'intervenir en conséquence; (ii) d'obtenir un consentement pleinement éclairé et volontaire; (iii) de protéger la vie privée des participants; et enfin, (iv) d'améliorer la transparence des puissants modèles d'apprentissage automatique afin de garantir une application éthique et impartiale dans le domaine des soins psychiatriques. Ce rapport identifie les défis qui en découlent ainsi que les questions éthiques non résolues en matière de santé mobile. Il formule également des recommandations sur la façon dont les chercheurs en santé mobile peuvent résoudre ces problèmes dans la pratique. À terme, nous espérons que ce rapport favorisera la poursuite des discussions portant sur les moyens de définir des méthodes de recherche adéquates pour la santé mobile en psychiatrie.

## Resumen

La aplicación de la tecnología digital a la investigación en psiquiatría está conduciendo rápidamente a descubrimientos y capacidades nuevas en el ámbito de la salud móvil. No obstante, el incremento de las oportunidades para recopilar pasivamente grandes volúmenes de información detallada sobre los participantes en los estudios, junto con los avances en las técnicas de estadística que permiten a los modelos de aprendizaje automático procesar tal información, ha planteado nuevos dilemas éticos relativos a los deberes de los investigadores: (i) hacer un seguimiento de los eventos adversos e intervenir en consecuencia; (ii) obtener un consentimiento voluntario plenamente informado; (iii) proteger la privacidad de los participantes; y (iv) aumentar la transparencia de los modelos potentes de aprendizaje automático para asegurar que puedan aplicarse de manera ética y justa en la atención psiquiátrica. En este análisis se destacan tanto los desafíos éticos nuevos como las cuestiones éticas aún sin resolver en la investigación sobre la salud móvil y se formulan recomendaciones sobre cómo los investigadores de la salud móvil pueden abordar dichas cuestiones en la práctica. En última instancia, se espera que este análisis facilite un debate continuo sobre cómo lograr las mejores prácticas en la investigación de la salud móvil dentro de la psiquiatría.

## ملخص

يؤدي تطبيق التكنولوجيا الرقمية على أبحاث الطب النفسي إلى اكتشافات وقدرات جديدة، بشكل سريع، في مجال الصحة عبر الهاتف المحمول. ومع ذلك، فإن زيادة فرص جمع كميات هائلة من المعلومات التفصيلية بشكل سلبي عن المشاركين في الدراسة، إلى جانب التطورات في التقنيات الإحصائية التي تسمح لنماذج التعلم الآلي بمعالجة هذه المعلومات، قد أدت لإثارة معضلات أخلاقية غير مسبوقة تتعلق بواجبات الباحثين من أجل القيام بما يلي: (1) مراقبة الأحداث السلبية والتدخل وفقًا لذلك؛ و(2) الحصول على موافقة طوعية واعية بالكامل؛ و(3) حماية خصوصية المشاركين؛ و(4) زيادة شفافية نماذج التعلم الآلي القوية وذلك لضمان إمكانية تطبيقها بشكل أخلاقي وعادل في الرعاية النفسية. تسلط هذه المراجعة الضوء على التحديات الأخلاقية الناشئة، والمعضلات الأخلاقية غير المحسومة في أبحاث الصحة عبر الهاتف المحمول، وتقدم توصيات حول كيف يمكن للباحثين، في مجال الصحة عبر الهاتف المحمول، معالجة هذه القضايا في الممارسة العملية. وفي النهاية، نأمل أن تؤدي هذه المراجعة إلى تسهيل النقاش المستمر حول كيفية تحقيق أفضل الممارسات في أبحاث الصحة عبر الهاتف المحمول في مجال الطب النفسي.

## 摘要

数字技术在精神病学研究中的应用正迅速带来移动医疗领域的新发现和新功能。然而，研究参与者被收集大量详细信息的机率增加，以及统计技术的进步，使机器学习模型能够处理此类信息，这就产生了关于研究人员职责的新型伦理困境：（i） 监测不良事件并进行相应的干预；（ii） 取得完全知情自愿的同意；（iii） 保护参与者的隐私；以及（iv） 提高强大的机器学习模型的透明度，以确保它们能够在精神病学保健中得到合乎道德和公平的应用。这篇文章强调了移动医疗研究中新出现的伦理挑战和未解决的伦理问题，并就移动医疗研究人员如何在实践中解决这些问题提出了建议。最终，我们希望这篇综述将有助于继续讨论如何实现移动医疗研究在精神病学中的最佳实践。

## Резюме

Использование цифровых технологий для проведения исследований в психиатрии способствует новым открытиям и появлению новых возможностей в сфере мобильного здравоохранения. Однако расширение возможностей пассивного сбора большого количества подробной информации об участниках исследования в сочетании с совершенствованием методов статистической обработки информации, позволяющих использовать модели машинного обучения для обработки такой информации, породило принципиально новые этические дилеммы в отношении таких обязанностей исследователей, как: (i) отслеживание нежелательных явлений и принятие соответствующих мер, (ii) получение полностью информированного добровольного согласия, (iii) защита конфиденциальности участников и (iv) повышение прозрачности мощных моделей машинного обучения, чтобы гарантировать их справедливое и этичное применение для оказания психиатрической помощи. В данном обзоре освещены возникающие этические проблемы и нерешенные этические вопросы исследований в области мобильного здравоохранения, а также содержатся практические рекомендации по их решению для исследователей в области мобильного здравоохранения. В конечном счете этот обзор должен посодействовать обсуждению вопроса применения наиболее совершенных практических методов для проведения исследований в психиатрии в рамках мобильного здравоохранения.

## Introduction

Mental illness affects approximately one in five persons in any given year and is the leading cause of disability worldwide.^1^^–^^3^ Nevertheless, there are approximately nine specialty care providers for those with psychiatric conditions per 100 000 persons across the globe,^4^ whereas approximately 17 600 per 100 000 experience a common mental illness,^1^ which suggests the current system is unable to deal with everyone needing treatment. In recognizing this fundamental issue of limited access, researchers have increasingly turned to technology to eliminate barriers to care.^5^ Indeed, approximately half of the world’s population has access to the internet and the average number of mobile or cell phone network subscriptions is greater than one per person globally.^6^^,^^7^ Consequently, technology-based treatments may offer innovative ways of closing gaps in access.

Mobile devices can provide unprecedented amounts of intensive, longitudinal data on movement intensity and duration, psychomotor disturbance, social interactions, concentration, sleep duration and quality, information-seeking behaviour and affective states.^8^ Moreover, the pattern-recognition capabilities of machine learning algorithms can be applied to these data to classify individuals by health-relevant characteristics (using, for example, defined “biomarkers” or “digital phenotypes”)^9^^,^^10^ and to predict clinical outcomes, such as a diagnosis or risk level.^11^ These classifications and predictions can then be employed to guide health-care assessments and to inform the delivery of individually tailored interventions. However, the application of machine learning approaches to individuals who may be members of vulnerable psychiatric population groups raises a variety of ethical dilemmas.^12^

## Ethical models and principles

Ethical models and principles, particularly those applied specifically to psychiatry, are central to our discussion of mobile health and machine learning in psychiatry research.^13^ Though ethical decision-making in health care is a vast field,^14^ we selected three well-known ethical models for consideration because of their relevance to decision-making in mobile health and machine learning: utilitarianism, Kantian ethics and principlism.

In utilitarianism (a form of consequentialism), actions that produce good consequences and benefit the largest number of people are prioritized, even if individuals’ privacy and autonomy must be sacrificed.^15^ This model may be applicable to several topics relevant to machine learning and mobile health, perhaps most obviously to balancing the right to privacy against the advancement of science. The Kantian model places less emphasis on the outcome of an action; rather, moral rules and ideals and rational principles are considered to be of the utmost importance in ethical decision-making.^16^ As applied to psychiatry research, this model might be especially relevant to researchers’ responsibility to act and monitor data collection. This approach contrasts with other ethical decision-making perspectives that emphasize the personal rights of individuals.^13^ These two models are particularly relevant to certain issues in machine learning and mobile health research, such as obtaining informed consent for monitoring and for accessing various data sources (e.g. smartphones, electronic health records and social media).

Finally, principlism is an ethical model that encompasses principles such as: (i) autonomy, which is defined as a patient’s right to choose their own course of action (e.g. with regard to interventions); (ii) beneficence (i.e. ensuring the patients being treated benefit); (iii) nonmaleficence (i.e. ensuring no unreasonable harm to patients); (iv) justice (e.g. fairness of, and access to, clinical practice); (v) confidentiality (i.e. ensuring the parties involved keep freely provided information confidential); and (vi) privacy (i.e. freedom from intrusion into personal matters).^14^^,^^17^^,^^18^ Each of these principles applies to the issues discussed below. In particular, we highlight the plethora of confidentiality and privacy issues that arise when the predictive algorithms applied in machine learning make use of patients’ data. Furthermore, autonomy, beneficence and nonmaleficence are relevant to the process of informed consent as well as to researchers’ responsibility to monitor data and possibly intervene to prevent harm to patients.

## Monitoring and intervening during data collection

Asking participants about sensitive topics (e.g. psychiatric symptoms) and assessing passive data streams (e.g. photographs on a smartphone) may result in researchers having information about negative health outcomes, such as worsening mental status, mood or physical health (i.e. adverse events). Researchers may have an ethical obligation to monitor the emergence of adverse events and potentially to intervene to mitigate negative outcomes. For example, in studies in which suicidal ideation and intent are being monitored, any indication of imminent risk may warrant taking safety measures. Researchers should consider the following when monitoring for adverse events: (i) the feasibility of monitoring and intervening given the resources and expertise available; (ii) validity (i.e. the possibility that monitoring or interventions may interfere with the behaviours under observation); and (iii) loss of privacy (e.g. breaking confidentiality when calling emergency services because of the suspected imminent risk of serious harm to oneself or others). The vastly increased frequency of data collection in daily life coupled to reduced personal contact with participants in mobile health assessment studies makes it more difficult to address these concerns than it would be in a traditional laboratory or clinic-based study.

### Feasibility

The drastic increase in the availability of information with no specific time frame or location has made it less straightforward and feasible to monitor risks and to intervene. For example, an individual may indicate the intention to end his or her life imminently, but researchers may be unaware of the individual’s location or be unable to make contact. Also, there is a lack of broad agreement about the threshold for initiating an intervention in response. Does the risk have be simply high or high and imminent? Should the intervention require a full assessment or be based only on study data? Should it ever be triggered by passively collected data (e.g. the content of text messages) or by predictive algorithms that are still under development? In some cases, institutional review boards advise collecting data only when the ability to monitor and intervene is viable; for example, in an anonymous online study, researchers may be advised not to ask for information about high-risk indicators (e.g. suicidal intent) or to collect open data streams (e.g. photographs) that might lead to an ethical obligation to intervene. However, this approach will limit researchers’ ability to learn about critical psychiatric phenomena as they occur in ecologically valid (i.e. real-world) settings and time frames.

### Validity

A key advantage of mobile health studies compared with laboratory or clinic-based studies is their greater ecological validity. However, close monitoring (and possible intervention) may alter participants’ responses, thereby compromising the validity of their data or increasing the risk they will withdraw from the study. For example, knowing that a study staff member may contact them in response to certain answers to survey questions could increase the likelihood that individuals who welcome such contact would give those answers. Others may be deterred from giving those same answers to avoid contact or being sent to hospital. Understanding how monitoring affects data validity is an important area of future research and is vital for developing guidelines on such issues.

### Privacy

There are several ethical questions about the use of study participants’ data to monitor, and intervene during, adverse events. For example, is it permissible to combine multiple streams of data to inform risk assessment (e.g. using physiological sensor data to assess reported health events) or to guide interventions (e.g. using smartphone geolocation data to deploy emergency services based on a self-reported response that indicates an imminent risk)? As in other contexts, currently there are no guidelines governing the trade-off between participants’ safety and their privacy, confidentiality and autonomy, especially when an intervention may occur without anyone speaking to the participant in advance. At the very least, researchers are encouraged to fully inform participants during the initial consent process about how their data will be monitored and used to guide emergency interventions.

## The right to privacy versus the advancement of science

Privacy considerations are especially germane to data collection that involves passive monitoring of participants’ daily lives, such as geolocation and actigraphy (i.e. movement) data. Since these data streams are collected without participants’ active engagement, there is a higher likelihood that they will lack full awareness of how such information can be transformed into a highly idiosyncratic and complex picture of a participant’s behaviour, which could potentially reveal an individual’s identity and actions. The ethical concerns that may arise from the tension between the advancement of science using mobile health technology and participants’ rights over their personal data are particularly relevant to: (i) informed consent, transparency and voluntary participation; and (ii) the protection of participants’ data (which could affect their security and safety).

### Informed consent

Informed consent is a key ethical requirement in research and has been defined as “the process by which a fully informed user participates in decisions about his or her personal data.”^19^ In obtaining consent, the following principles must be taken into account: (i) disclosure, whereby researchers clearly and thoroughly inform prospective participants of the potential risks and benefits of participating in the study; (ii) agreement, whereby participants are asked to accept or decline participation; (iii) comprehension, wherein participants must demonstrate full and detailed understanding of the study; (iv) competence (i.e. the participant must have the mental and physical ability to provide consent); and (v) voluntariness (i.e. participants consent of their own volition).^17^^,^^18^

Mobile health research using machine learning presents unique challenges in adhering to these principles. Recently, several recommendations have been made in response to the growing challenge of obtaining informed consent in the modern digital world.^20^^,^^21^ One of the most difficult principles to address is comprehension because consent is often requested online.^22^ Currently, digital platforms frequently obtain consent via terms-of-service agreements, which are written in incomprehensible legalese and are rarely even read.^23^^,^^24^ Researchers have an ethical responsibility to minimize the risk of this occurring by disclosing all relevant study information in a comprehensible manner. To enhance comprehension, the overall consenting process should be thorough, engaging and accessible. Giving consent in person or through a video or phone call is preferable, but is often not feasible for large-scale, mobile health projects. Researchers should carefully consider the potential trade-offs between how consent is obtained and maximizing access to study benefits or interventions, particularly in low-resource areas where individuals may have few options for good-quality care of their psychiatric conditions. When consent is obtained via an online platform, the process should highlight key information and prevent participants from clicking through without reading.^25^ Engagement can be increased by using interactive screens and video or audio content and by summarizing sections in clear, concise language.^23^^,^^26^ Participants’ comprehension and competence can both be assessed using short quizzes or games and a live chat feature can allow participants to clarify their understanding.^21^

The principle of voluntariness implies that engagement in research is noncoercive and of the participant’s own free will and that participants have been presented with an explicit opportunity to decline participation. These criteria can be enhanced by giving participants the opportunity to consent to different data collection streams (e.g. to opt into daily diaries but not geolocation data) or to different research modules (e.g. to opt into the cardiovascular health module but not the sexual dysfunction module).^21^ Although this approach maximizes voluntariness and the autonomy of each participant, it may come at the cost of sacrificing data quality and, thereby, predictive accuracy. For instance, if participants opt out of entire modules, it could be more difficult to accurately determine their health risk levels and to deliver optimized, just-in-time interventions, which may rely on algorithms that require multiple data streams (e.g. the cross-referencing of geolocation and accelerometer data that could predict drug use in a high-risk environment). Therefore, in addition to disclosing the risks of opting in to all data streams, researchers and health providers should help participants to make informed decisions by fully revealing the personal benefits of opting in and the societal benefits of each research module. In addition, obtaining ongoing consent is recommended: participants should repeatedly revisit the terms of the study protocol throughout its duration to ensure their continued comprehension, competence and voluntary consent.^27^

### Protection of participants’ data

Ethical considerations also extend into the realm of protecting participants’ data. Since mobile health research involves the passive collection of data that is not typically associated with health outcomes (e.g. screen time) and may fall outside long-standing regulatory protections, researchers must implement the highest of ethical standards in protecting such intensive and potentially sensitive data. Moreover, highly detailed information is often collected from multiple sources and it is possible that combining data streams could produce information that could identify individuals and be used in unauthorized ways (thereby impacting an individual’s employment opportunities or eligibility for insurance) or that could be commercialized for targeted advertisements. In extreme circumstances, unauthorized use of such highly granular data could put a participant’s safety at risk if accessed by ill-intentioned actors.

Some recent efforts to establish regulations to protect personal data are the European Union’s General Data Protection Regulation and California’s Consumer Privacy Act, both of which give people more access to, and autonomy over, their data, require greater transparency on data use and stipulate tighter oversight to ensure the protection and security of data.^28^^,^^29^ By following the recommendations of these regulatory initiatives, research teams can adopt dissociable roles for the handling and processing of intensive mobile health data. For example, one individual could be designated to manage identifying information, whereas another could handle unidentifiable aggregated data and conduct analyses. This would enhance data security by minimizing the proliferation of sensitive information. An additional security step could involve further dissociation of roles: face-to-face interactions could be carried out by yet another individual who is blinded to highly granular personal information (e.g. home addresses), thereby mitigating risks to participants’ safety.

Shifts towards research involving intensive, mobile health data and open access data-sharing provide unprecedented opportunities for growth in translational research. To guard against the unintended consequences of these advances, researchers should consider adopting novel models for participants’ consent, data protection and engagement in the research process.^30^^,^^31^ The recommendations outlined here point to a participant-centric model that could maximize protection for study participants while supporting research aimed at improving psychiatric outcomes.

## Machine learning models: performance versus interpretability

Machine learning is being increasingly applied in psychiatry for diagnosis, treatment selection and clinical administration.^32^^,^^33^ However, its future is affected by a key ethical dilemma associated with the trade-off between the performance and the interpretability of machine learning models. Interpretability relates to the ease of deciphering how a set of inputs to a model (e.g. patient characteristics and medical history) result in a particular output or prediction (e.g. a diagnosis or risk assessment). More complex models (e.g. random forests and neural networks) often have greater accuracy, but lower interpretability than simpler models (e.g. naïve Bayes classifiers).^34^ From a clinical perspective, models with low interpretability could raise ethical challenges because it may be difficult to understand how input variables contribute to the model’s predictions. Moreover, given that a model’s predictions could have substantial clinical consequences, such as hospitalization or the administration of medication, its accuracy is vital.

Machine learning models are predominantly conceptualized as support tools and not as replacements for clinicians.^35^^,^^36^ However, it is not clear whether or how these models should be incorporated into clinical practice. For example, a prognostic model with high predictive accuracy, but low interpretability might result in a clinician knowing a patient is at risk, but not what to target with an intervention. In addition, how clinicians should share information from machine learning models with patients also gives rise to ethical questions. Would patients want to know they are at risk, particularly if they cannot be told why (as factors included in machine learning models generally cannot be interpreted as having a causal impact on outcomes)? Sharing information from an uninterpretable model may adversely affect a patient’s conceptualizations of their own illness, cause confusion and prompt concerns about transparency.^37^^,^^38^ So far, research suggests there is no clear consensus among patients on whether they would want to know this kind of information about themselves,^37^ which leaves psychiatrists to balance the potential utility of a machine learning model’s predictions against the risk of liability and the patient’s reactions.^39^

Furthermore, when a model is not interpretable, a clinician’s ability to be cognizant of possible fairness issues could be limited.^40^ In machine learning, fairness encompasses concerns about how data-driven approaches can reflect and perpetuate biases rooted in social inequality and discrimination.^41^^,^^42^ A model’s predictions can vary systematically across demographic groups if, for example, the data being sampled reflects societal inequalities (i.e. historical bias) or if the sampling methods result in the underrepresentation of certain groups (i.e. representation bias).^43^ Consider a machine learning model trained using the electronic health records of medical visits;^44^ this model might not be able to accurately predict psychiatric conditions in immigrant populations that avoid interacting with the health-care system.^45^ Additionally, clinician bias in the International Classification of Disease’s codes or clinical notes can introduce variations in the inputs to a machine learning model that, in turn, bias the model’s predictions for minority groups.^46^ Further, with less interpretable models, it can be more challenging to detect, track and rectify these different sources of bias.

Although numerous ways of addressing issues of effectiveness and fairness in machine learning are emerging,^47^ these are often based on one-time analyses of a single data set. At the forefront of machine learning today, the trend is to allow machine learning to change iteratively over time with each new piece of incoming data. However, the practice of employing continually adaptive algorithms raises questions on how often algorithms should be updated and when reassessment is warranted. Recently, the United States’ Food and Drug Administration proposed new regulations for monitoring changes in adaptive algorithms as they continuously learn from real-world data.^48^ These regulations require manufacturers to prespecify the changes they anticipate because of online learning and the protocols in place for addressing risks that might result from changes to an algorithm’s operations. Assessments of potential risk consider the degree to which an algorithm contributes to the psychiatric decision (e.g. guiding treatment versus assigning a diagnosis) and the severity of the patient’s condition (e.g. identifying individuals at risk for developing a psychiatric disorder in the future, versus identifying those at an acute risk for suicide). The emphasis is on transparency, manufacturers should tell end-users about the changes occurring in algorithm performance over time and provide transparent information about algorithm processes in a way that enables clinicians and patients to engage in meaningful risk assessments of the machine learning model. Less interpretable models constrain transparency and thus limit potential contributions from all important stakeholders. Instead of focusing on how machine learning can be used in clinical care within psychiatry, the priority might be first to consider whether predictions from a specific machine learning model are appropriate for informing decisions about a particular intervention.^49^

## Conclusions

This review highlights the wide range of ethical issues faced by psychiatry researchers in the digital age. Although mobile health and machine learning have the potential to facilitate great advances in, and close access barriers to, psychiatric research and care globally, they also give rise to new ethical questions concerning, for example: (i) the responsibility to monitor naturally occurring adverse events and intervene accordingly; (ii) the need to guard privacy rights and ensure informed consent while conducting scientific research; and (iii) the importance of increasing the transparency of powerful machine learning models to ensure they can be applied ethically and fairly in clinical decision-making. Ultimately, the issues need to be thoughtfully considered by several stakeholders, including regulatory agencies, clinicians, participant-advocacy groups and ethicists, as well as researchers. As the number of mobile health studies increases and mobile technologies evolve,^50^ it will only become more critical to establish consensus-based guidelines for ethics in mobile health research.
